# Use of a Temporary Pacemaker During Cesarean Section in a Pregnant Patient With Neurally Mediated Syncope: A Case Report

**DOI:** 10.7759/cureus.97291

**Published:** 2025-11-19

**Authors:** Shun Takaki, Fumiya Sawasaki, Keiko Honma, Kan Takahashi

**Affiliations:** 1 Anesthesiology, Kanazawa Medical University, Uchinada, JPN; 2 Anesthesiology, Shiga University of Medical Science, Otsu, JPN

**Keywords:** bradycardia, cesarean section, combined spinal and epidural anesthesia, neurally mediated syncope, temporary pacemaker

## Abstract

A 39-year-old pregnant woman with a history of neurally mediated syncope (NMS) and a documented eight-second sinus arrest on her Holter electrocardiography (ECG) was scheduled for cesarean delivery. A temporary pacemaker (PM) was inserted preoperatively to prevent severe bradycardia. Combined epidural and spinal anesthesia (CESA) was carried out. Intraoperatively, severe bradycardia triggered PM activation. Prompt administration of ephedrine and atropine sulfate stabilized her vital signs, and the PM was deactivated within two minutes. The cesarean section proceeded without further complications, and both mother and neonate had favorable outcomes. The PM was removed on postoperative day 2, and no syncope episodes were observed during hospitalization or follow-up. This case demonstrates the effectiveness of temporary PM use in preventing severe bradycardia during cesarean section in a patient with NMS. Multidisciplinary planning and individualized hemodynamic management are essential for achieving safe delivery outcomes in high-risk pregnancies complicated by reflex syncope.

## Introduction

Neurally mediated syncope (NMS) is characterized by a transient loss of consciousness without any apparent abnormalities in the cardiovascular or central nervous systems. This condition encompasses vasovagal syncope (VVS), situational syncope, emotional syncope, and reflex syncope [1]. While the pathogenesis of NMS is complex, excessive activation of the vagal reflex, leading to a sudden drop in blood pressure and heart rate, is considered a primary factor [2]. In daily life, NMS may occur in response to mental or physical stress, prolonged standing, or pain [3].

During labor, the combination of pain, fear, and anxiety increases the risk of NMS. Pregnancy complicated by NMS carries increased risks during delivery due to physical and psychological stress, as well as hemodynamic fluctuations caused by bleeding and pain stimuli [4]. Maternal hemodynamic instability associated with NMS, such as hypotension and bradycardia, can threaten fetal well-being and long-term prognosis. There are few reports on the use of temporary pacemakers (PMs) to manage high-degree atrioventricular block in pregnancy; however, cases of their use for NMS are extremely rare [5].

This report describes a case of a pregnant woman with NMS who underwent cesarean delivery with the placement of a temporary PM for hemodynamic stability.

## Case presentation

The patient was a 39-year-old woman with NMS who was referred to our obstetrics and gynecology department for perinatal management. The patient, gravida 5 para 3, had experienced delayed emergence from sedation during surgery for a missed miscarriage, which became a traumatic event leading to complex post-traumatic stress disorder (PTSD), a condition that shares with NMS a common pathway of autonomic instability whereby emotional or physiological stressors can trigger reflexive cardiovascular responses. Preoperative tests revealed no abnormalities. A 12-lead electrocardiogram showed a sinus rhythm at 55 bpm.

**Figure 1:**
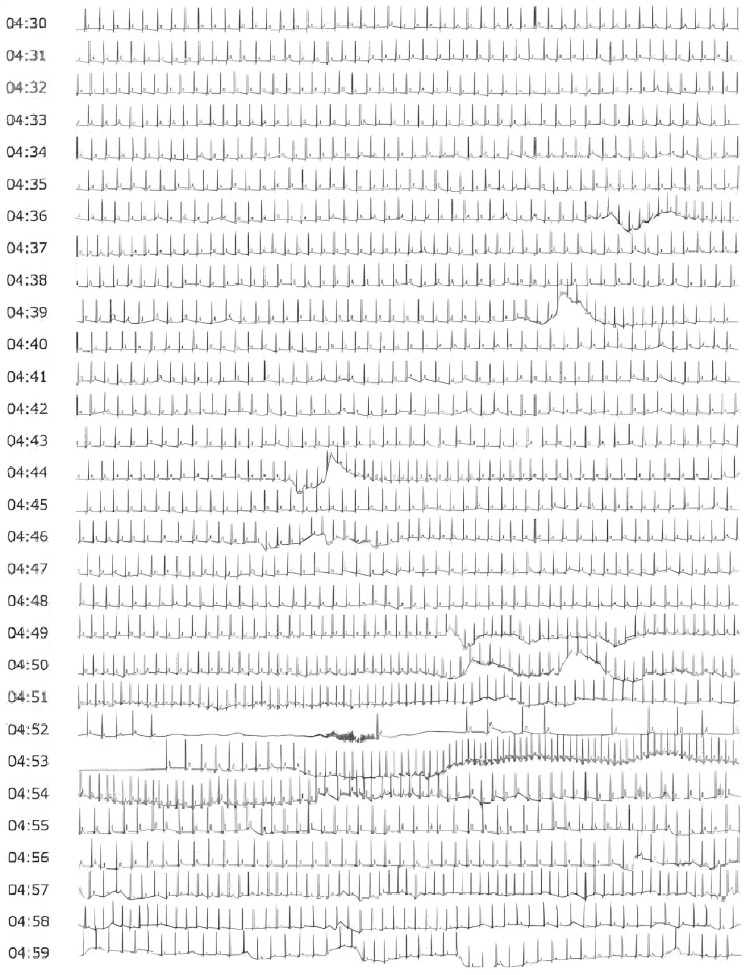
The Holter ECG recorded from 4:30 a.m. to 4:59 a.m. Continuous Holter monitoring obtained between 4:30 a.m. and 4:59 a.m., demonstrating the patient’s sinus arrest from 4:52 a.m. to 4:53 a.m.

**Figure 2:**
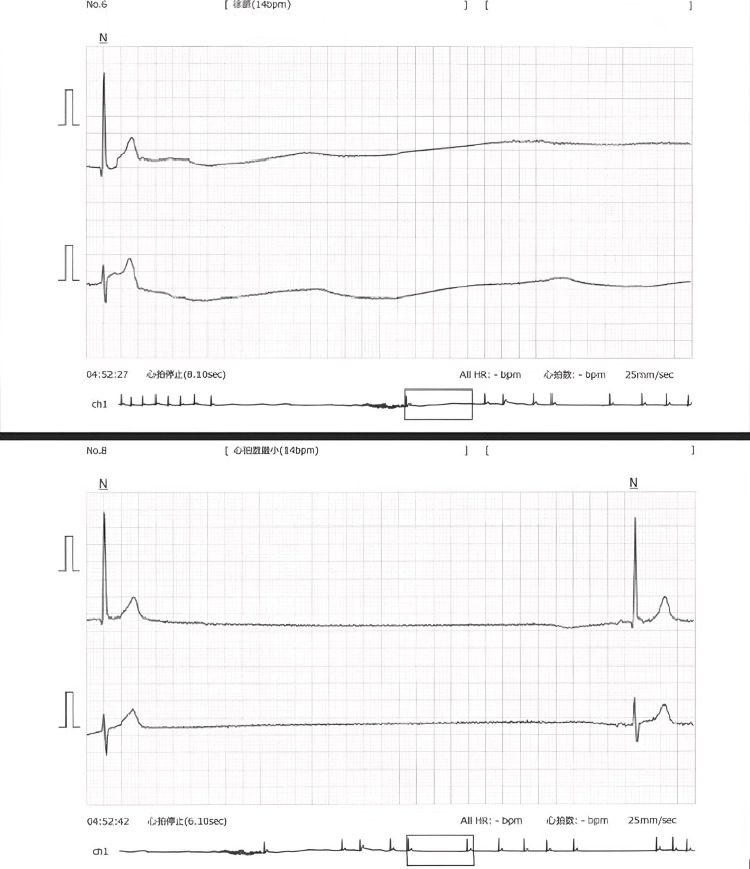
Enlarged Holter tracings during sinus arrest. Eight-second sinus arrest was recorded in the Holter ECG.

Her history of syncope began in childhood, with manageable episodes accompanied by prodromal symptoms, and only a few occurrences after school age. At age 29, she experienced nausea as a prodromal symptom during vaginal delivery, but her subsequent deliveries at ages 30 and 34 were uneventful. At the age of 35, while undergoing Holter electrocardiography (ECG) monitoring, she developed severe chest pain and nausea following early-morning abdominal pain, culminating in syncope. The Holter ECG recorded an eight-second sinus arrest, resulting in a diagnosis of NMS (Figures 1-2). She avoided further syncopal episodes through lifestyle management that involved taking a moment to pause before initiating any action. However, the current pregnancy was classified as high risk for recurrent syncope triggered by the stress of labor. After thorough consultation, it was decided to place a temporary PM and proceed with a cesarean section to maintain hemodynamic stability during delivery.

**Figure 3:**
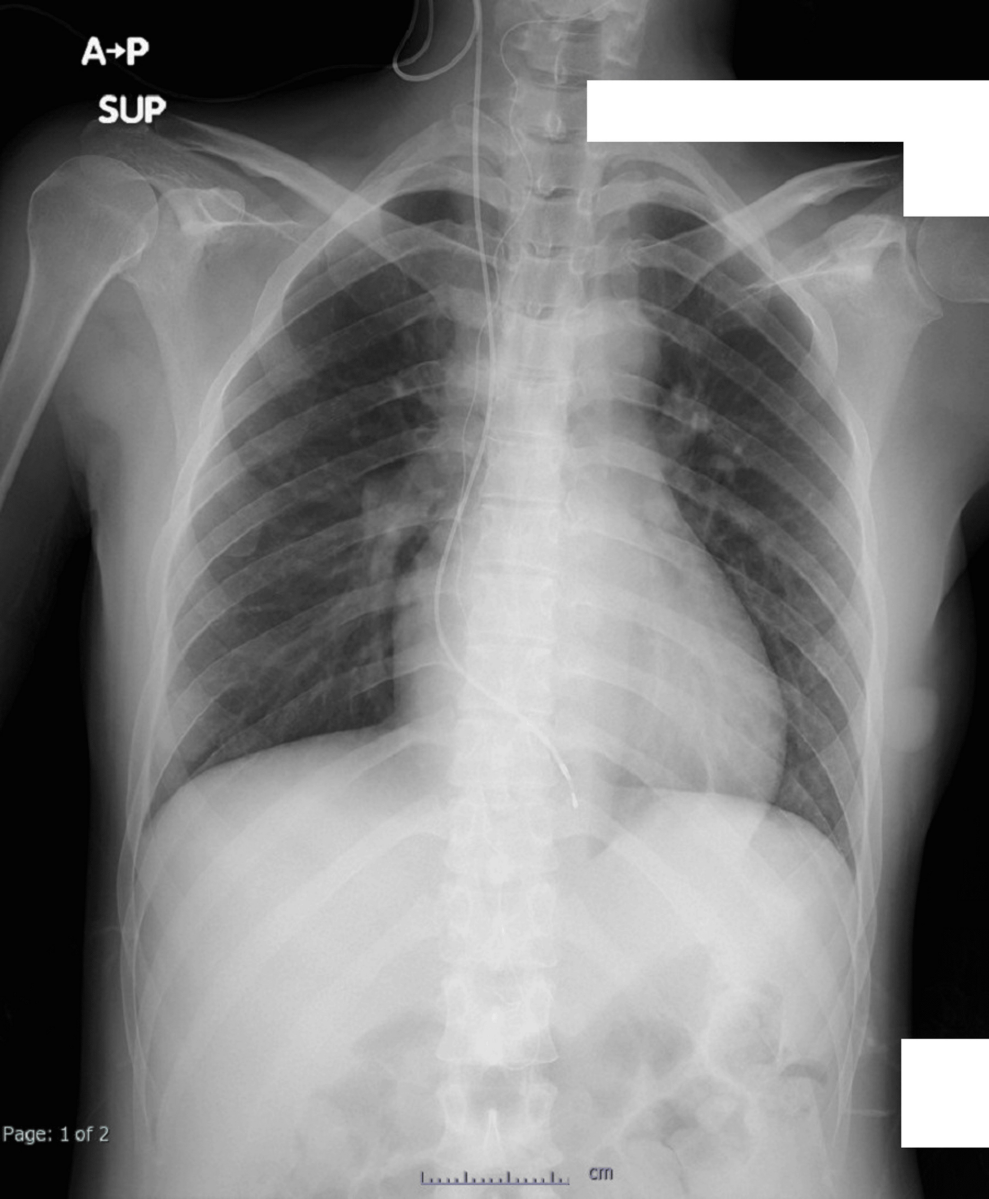
Chest X-ray taken after temporary pacemaker was inserted via right internal jugular vein to the right ventricle.

After providing the patient with a preoperative explanation of general anesthesia and combined epidural and spinal anesthesia (CESA), CESA was chosen due to the patient’s concern about the potential effects of anesthetic agents on the fetus and her desire to be awake to see her baby immediately after delivery. On the day of surgery, a temporary PM was inserted via the right internal jugular vein and set to VVI mode at a pacing rate of 40 bpm (Figure 3). Upon entering the operating room, the patient's heart rate was 55 bpm, indicating bradycardia. Epidural anesthesia was initiated with catheter placement at the Th12/L1 interspace, followed by a test dose of 3 mL of 1% lidocaine, which showed no abnormal findings of intrathecal placement. Spinal anesthesia was then administered at the L3/4 interspace using 2 mL of 0.5% isobaric bupivacaine and 20 mcg of fentanyl. Ten minutes later, sensory block levels were confirmed at Th4 on the right and Th6 on the left.

**Figure 4:**
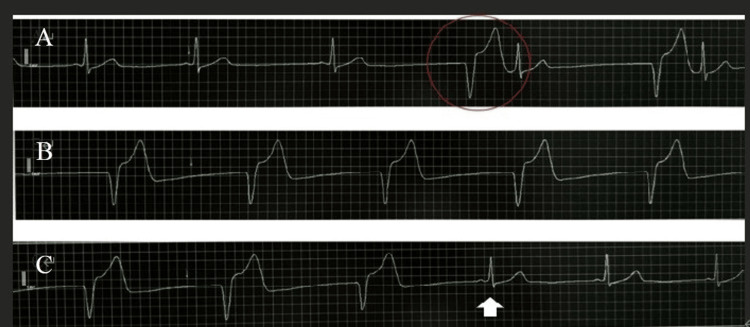
Intraoperative electrocardiographic monitoring. (A) Sinus bradycardia with subsequent pacemaker activation, demonstrated by pacing spikes followed by ventricular capture (red circle). (B) Complete pacemaker dependency showing regular pacing spikes and broad QRS complexes. (C) Recovery of intrinsic sinus rhythm with disappearance of pacing spikes and restoration of narrow QRS complexes (white arrow).

**Figure 5:**
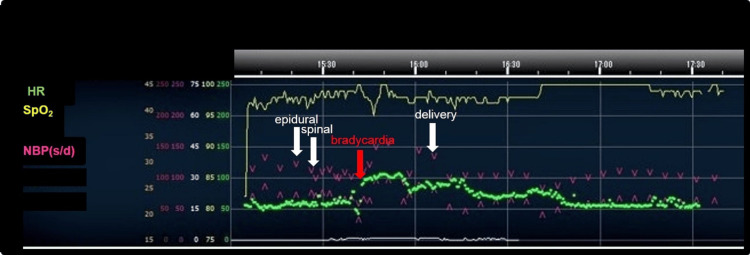
Intraoperative hemodynamics.

Five minutes later, sudden bradycardia occurred, activating the PM (Figures 4, 5). Ephedrine (8 mg) and atropine sulfate (0.5 mg) were administered immediately. After approximately two minutes, the PM ceased activity. The patient maintained consciousness during this time. Reassessment of the sensory block revealed an extension to Th3 bilaterally. Although nausea persisted until delivery of the neonate, the surgery concluded without further complications. Apgar scores were 8 and 9.

Postoperative pain management was achieved using patient-controlled epidural analgesia with 0.25% ropivacaine (80 mL) combined with 170 mL of normal saline. After returning to the recovery room, the patient occasionally experienced PM activation during sleep; however, her baseline sleep heart rate was typically in the high 30s bpm, and her pain was well-controlled. The temporary PM was removed on postoperative day 2.

Throughout the postoperative course, no episodes of syncope were observed. The patient was discharged home on postoperative day 7. She was subsequently evaluated in our outpatient clinic for 23 days post-discharge, with both mother and infant showing no issues. The patient has continued regular follow-up with her local physician.

## Discussion

Syncope during pregnancy has been examined in several large cohort studies, demonstrating a prevalence of approximately 0.3-1% and associations with adverse neonatal outcomes, including preterm birth, intrauterine growth restriction, congenital anomalies, and long-term neurological morbidity in the offspring [6,7]. In contrast, data specifically focusing on syncope during labor are scarce. Nevertheless, maternal syncope or pronounced hypotension during labor and cesarean delivery can reduce uteroplacental perfusion and has been associated with intrapartum fetal heart rate abnormalities and lower umbilical arterial pH, indicating neonatal acidemia [8]. These findings highlight the importance of careful hemodynamic monitoring and proactive management strategies during delivery, especially in patients with a history of NMS.

According to the 2017 American College of Cardiology/American Heart Association/Heart Rhythm Society (ACC/AHA/HRS) Guideline, VVS is the most common cause of syncope. The effectiveness of drug therapy is modest. Patient education on the diagnosis and prognosis is recommended (Class I). Dual-chamber pacing might be reasonable in a select population of patients over 40 years of age with recurrent VVS and prolonged spontaneous pauses (Class IIb) [9].

While patient education approaches are effective in daily life, they may be insufficient under the physical and emotional stress of pregnancy and delivery. In the present case, the patient had a history of syncope due to NMS and an eight-second sinus arrest documented by Holter ECG. Careful hemodynamic management during delivery was crucial. The use of a temporary PM allowed a rapid response to intraoperative bradycardia, preventing severe circulatory collapse. Early intervention with ephedrine and atropine sulfate ensured prompt recovery of hemodynamic stability.

Although reports on temporary PM use for patients with reflex syncope are scarce, its application during labor has significant preventive value. Anesthesia management also requires particular attention. NMS patients are prone to exaggerated vagal reflexes, and sympathetic blockade from spinal anesthesia may exacerbate vasodilation and bradycardia, increasing the risk of severe bradycardia or cardiac arrest [10]. Furthermore, the Bezold-Jarisch reflex may also be triggered by spinal anesthesia, which can cause hypotension and bradycardia [11]. CESA offers good anesthesia and analgesia for cesarean delivery, but may cause profound hypotension and bradycardia in NMS patients due to sudden sympathetic blockade. Gradual induction with low-dose epidural anesthesia, vasopressor support, and preparation for pacing are advisable. In contrast, general anesthesia, by suppressing autonomic reflexes, may reduce cardiovascular events and is preferable in patients with severe anxiety or PTSD. Because PTSD and NMS share autonomic instability, emotional stress can trigger exaggerated reflex responses. In this case, CESA was chosen based on the multidisciplinary coordination and the patient’s desire to remain awake for immediate skin-to-skin contact after delivery. To mitigate the adverse events of sympathetic blockade caused by CESA, the present patient received a temporary PM and underwent vigilant intraoperative hemodynamic monitoring, enabling immediate intervention for bradycardia. This approach effectively stabilized the patient's unique circulatory vulnerabilities and ensured safe anesthesia and surgical progression.

In this case, multidisciplinary planning among anesthesiologists, obstetricians, and cardiologists ensured hemodynamic stability. Preoperative discussions identified anxiety triggers and established coping strategies, while continuous reassurance by the nursing staff minimized stress responses. The patient had comorbid PTSD, which may amplify autonomic instability. Incorporating psychological support or anxiolytics preoperatively may further optimize care in similar patients [12]. For future cases, earlier involvement of mental health professionals and simulation-based team preparation could further improve management and patient outcomes. Such collaborative strategies should be encouraged in managing high-risk obstetric cases with autonomic dysfunction. 

## Conclusions

This case demonstrates the efficacy and safety of temporary PM placement in the perinatal management of patients with NMS. Proactive insertion of a temporary PM enabled prompt treatment of intraoperative bradycardia and contributed to favorable maternal and neonatal outcomes. Individualized anesthetic planning and multidisciplinary coordination are essential in managing such high-risk pregnancies. Accumulating additional case reports will help establish clearer, evidence-based guidelines for perioperative PM use and identify patients who may benefit most from this preventive strategy. Given the limited number of published cases, further accumulation of similar clinical experiences is essential to refine the indications for PM use, determine optimal perioperative management strategies, and establish evidence-based guidelines for pregnant patients with NMS. Future studies should also aim to identify patient characteristics that predict the greatest benefit from prophylactic pacing, thereby improving safety and consistency in the management of this rare but challenging condition.
